# Beyond the GFAP-Astrocyte Protein Markers in the Brain

**DOI:** 10.3390/biom11091361

**Published:** 2021-09-14

**Authors:** Agnieszka M. Jurga, Martyna Paleczna, Justyna Kadluczka, Katarzyna Z. Kuter

**Affiliations:** Department of Neuropsychopharmacology, Maj Institute of Pharmacology, Polish Academy of Sciences, 31-343 Kraków, Poland; jurga@if-pan.krakow.pl (A.M.J.); paleczna@if-pan.krakow.pl (M.P.); kadlucz@if-pan.krakow.pl (J.K.)

**Keywords:** astroglia, reactive astrogliosis, astrocyte reactivity, astrocyte phenotype, astrocyte state, protein markers

## Abstract

The idea of central nervous system as one-man band favoring neurons is long gone. Now we all are aware that neurons and neuroglia are team players and constant communication between those various cell types is essential to maintain functional efficiency and a quick response to danger. Here, we summarize and discuss known and new markers of astroglial multiple functions, their natural heterogeneity, cellular interactions, aging and disease-induced dysfunctions. This review is focused on newly reported facts regarding astrocytes, which are beyond the old stereotypes. We present an up-to-date list of marker proteins used to identify a broad spectrum of astroglial phenotypes related to the various physiological and pathological nervous system conditions. The aim of this review is to help choose markers that are well-tailored for specific needs of further experimental studies, precisely recognizing differential glial phenotypes, or for diagnostic purposes. We hope it will help to categorize the functional and structural diversity of the astroglial population and ease a clear readout of future experimental results.

## 1. Introduction

Yesterday, astrocytes were just supportive allies in the central nervous system (CNS), and today we witness remodeling of the knowledge about them from the top to the bottom. New nomenclature and awareness regarding astroglial heterogeneity and the functional polarization spectrum is being proposed, and consequent changes in astroglial markers’ usefulness need to be reorganized.

Glial cells, underestimated for a long time, now are gaining proper interest. Their active role in brain functioning is finally noted. Astrocytes have enormous supportive potential towards neurons and can proliferate through the whole life-span despite aging, which makes them a perfect therapeutic target for multiple CNS diseases. These glial cells have multiple essential roles in the brain’s functioning and homeostasis, which are also reflected in their heterogeneity [1,2]. Nevertheless, it is important to remember that astroglia can also act in either a cytotoxic or cytoprotective way, depending on their current activation phenotype. The factors they express directly relate to their functional state. Therefore, it has become an urgent issue to precisely recognize various astrocyte phenotypic states via more specific markers.

In this review, we will focus on the physiological and pathological markers of particular functions and phenotypes of astrocytes, which could be helpful in precise identifying of their states in research to define mechanisms of their action, but also could be used as diagnostic markers in CNS diseases (Table 1).

### Astrocyte Functions in the Brain 

**Figure 1:**
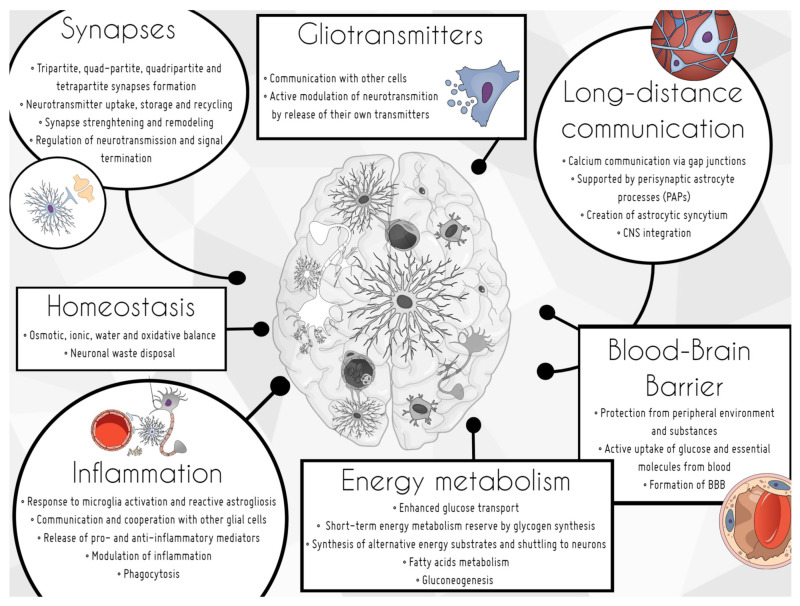
Astrocyte functions in the brain.

Astrocytes are the predominant non-neuronal cellular population in the CNS (approximately 50%) and the most diverse phenotypically [3]. The overriding role of these cells which cannot be underestimated is their support for neuronal development and general well-being. Astrocytes maintain tissue homeostasis and can be considered as supportive in many contexts, such as structure, energy metabolism, trophic factors delivery, synaptic transmission, long-distance communication, and inflammation (Figure 1). The excellence of astroglial multitasking is underlined by the fact that a single cell can enwrap from 100 thousand to 2 million synapses, as measured in rodents and humans respectively [4].

Morphologically, astrocytes consist of soma and processes, which can be divided in descending order into branches, branchlets, and leaflets. They communicate with other cells, synapses, and blood vessels [5]. The well-known and important structure is a tripartite synapse built with three equally relevant pieces: pre- and postsynaptic neuronal membranes, and an astrocyte process. There are even more specified units proposed. The ‘quad-partite synapse’ is a term proposed by Schafer et al. in 2013 to describe the astrocyte–microglia–glutamatergic neuron unit [6,7]. The ‘quadripartite synapse’ was described by Syková et al. for presynaptic and postsynaptic neuronal terminals, astroglia processes, and the extracellular space with its content [8]. The ‘tetrapartite synapse’ includes specifically an extracellular matrix (ECM) instead of a whole ECS, proposed by Dityatev et al. [9]. It shows that not only neurons but also glia and their environment are partners in the synapse. Astroglial endfeet are also key players in regulation of CNS extracellular fluid content thanks to their formation of the blood–brain barrier (BBB), a physical and functional filter, along with endothelial cells and pericytes [10]. One of the astroglial functions is to maintain homeostasis by regulation of water, ions, oxidative state, metabolic waste, and toxins [11].

Structural support for neurons is crucial in CNS formation, when astrocytes guide developing neurons’ traffic in layers of the brain cortex. They serve as scaffolding for migrating neuroblasts in physiological in vivo conditions; and there were attempts to mimic this ability to elongate in vitro for regenerative purposes [12,13]. They also support neurogenesis in adulthood by neurotrophin release and may serve as progenitor cells after, i.e., traumatic CNS injury [14]. By regulating formation, maintenance, and elimination of synapses, astrocytes are responsible for local structural remodeling and neuronal circuits’ integrity. 

Astrocytes maintain the functional neurotransmission in the synaptic cleft by fast reuptake of neurotransmitters and terminate the signal transduction using specific transporters (e.g., dopamine transporter, DAT; norepinephrine transporter, NET). One of the major excitatory neurotransmitters, glutamate, can be neurotoxic if released in excess. Using excitatory amino acid transporters 1 and 2 (EAAT1 and EAAT2, also known as GLAST and GLT-1, respectively, in rodents) astrocytes accumulate 80% of glutamate from the synaptic cleft to prevent its toxic effects. Astrocytes react to neurotransmitters with specific receptors and communicate with other cells by releasing gliotransmitters (e.g., glutamate, D-serine, and ATP). They communicate with each other also by calcium signals which can be transferred through gap junctions to multiple cells, creating an astroglial syncytium. Such a cellular network created by astrocytes enables them to communicate over larger distances (for review see [15]). 

Optimal tissue oxidation status is kept by synthesis and shuttling of antioxidants (e.g., ascorbate, glutathione) by astrocytes. The neuronal metabolic by-product, ammonia, is removed and utilized also by these cells. Another important astrocytic function is regulation of local energy metabolism, which is a feature distinguishing them from other CNS cell types. Due to the endfeet’s proximity to the BBB, astrocytes transport the main brain energy fuel, glucose, from the blood. They also synthesize and shuttle energy substrates for neuronal metabolic processes (glucose, ketone bodies, lactate) and synchronize their uptake from blood vessels with neuronal needs. Astrocytes also serve as the only documented brain energetic reserve by synthesizing glycogen [16]. 

In contrast to microglia, standing in the first line of the inflammatory response process, astrocytes are not as well equipped with receptors recognizing pathogens, but they can also become reactive, release inflammation mediators or even phagocyte unwanted materials [17]. Astrocytes strictly cooperate with other glial cells in the inflammatory response and actively modulate it. 

## 2. General Astrocyte Markers

Astroglia protein markers can be expressed as membrane, intracellular, or secreted factors. Among them are structural proteins, membrane channels and transporters, transcription factors, as well as energy metabolism markers. Not all of them are selective only to astrocytes. Sometimes it is just a matter of expression level when compared to the other cell groups and between heterogenic astrocyte subpopulations. Importantly, the particular set of markers and their amount strongly depends on the physiological condition of the cell and its activation phenotype. It is reasonable to use plural markers for the best readout of research results. Here, the general markers of astrocytes will be discussed whose expression is present in the majority of astrocyte subtypes and cell states (Table 2). 

The everlasting trio used for astroglia recognition rises above divisions and will open this paragraph. The astrocytic structural marker protein is a glial fibrillary acidic protein (GFAP) present in the majority of CNS astrocytes, although expressed diversely in cells from different brain regions, as well as in the neuronal stem cells (see below in “Heterogeneity of astrocytes” paragraph) [18,19]. GFAP is an intermediate filament (IF) type III protein found under different isoforms (α, γ, δ/ε, and κ in human brain, β additionally in rodents). Isoform α is the most abundant. GFAP’s general function is to support astrocytes and the BBB structure mechanically [20,21]. It labels mainly white matter astrocyte extensive branching and slightly their cell body [3,22]. This makes it the best marker for studying complex astrocytes’ morphology. The cytosolic protein S100β labels cell bodies of small astrocytes with less extended branching, and preferentially marks gray matter cells complementing GFAP staining [3,22]. Last but not least, the folate enzyme aldehyde dehydrogenase 1 family member L1 (ALDH1L1) converts NADP to NADPH in the adult brain and was found to be a reliable selective astrocytic protein [23,24,25]. Below, you can find complementary marker proteins, which are beyond the classics.

### 2.1. Structural Proteins

One of the most commonly used astrocyte markers is vimentin, IF III, which is structurally similar to GFAP, although considered rather as a pan-glial marker and can be found also in fibroblasts, endothelial cells, macrophages, neutrophils, and lymphocytes [26,27]). In the normal adult CNS, vimentin is mostly expressed in some specialized glial cells such as Bergmann and radial glia or ependymal cells [28]. Its amount decreases significantly after brain development, when it becomes only detectable in cell bodies. Expression in radial glia and immature astrocytes proves its role in differentiation and maturation of astrocytes [28]. Vimentin immunoreactivity is normally very low but also increases after activation, for example, due to injury [29]. Nestin is considered to be a marker of stem or progenitor cells but it is also variably expressed in developing astrocytes and much less abundant in mature astrocytes. It is present mainly in the cytoplasm and much less in the processes [30]. An intermediate filament protein called transitin is transiently expressed by radial glia during CNS development. This protein is expressed by midline radial glia structures, by several axon commissures, and by Bergmann glia of the developing cerebellum [26]. Synemin, another IF, is co-expressed by immature astrocytes along with GFAP, vimentin, and nestin [31]. Ezrin and radixin are structural markers of fine, peripheral astrocyte processes [32]. Other proteins with functions in actin-related processes enriched in astrocytes are calponin-1 (CNN1), formin-2 (FMN2), LIM zinc-binding domain-containing Nebulette (NEBL), PDZ, and LIM domain protein 7 (PDLIM7) or synaptopodin-2 (SYNPO2) [33]. 

### 2.2. Transcription Factors

Among many functions, transcription factors control the initiation of specific cell-type differentiation. Some of them can be used as astrocytic markers. The SRY-related high mobility group (HMG) box gene 9 (SOX9) is a transcription factor localized in cell nucleus, expressed almost exclusively in astrocytes [34]. It serves as a good astroglia marker in the adult brain, outside the neurogenic zones. Astrocytes are also significantly enriched in NK homeobox gene-coded proteins, from which NKX3-1 and NKX6-1 were identified in the brain as astrocytic region-specific transcription factors, and are exclusively expressed in astrocytes in the olfactory bulb and brainstem, respectively [35,36]. Another protein of this family, NKX2-1, binds to the GFAP promoter and regulates its expression. It controls astroglia production spatiotemporally in embryos by regulating stem cell division and specification of the precursors in telencephalon, but also cell differentiation of telencephalic GABAergic interneurons and oligodendrocytes [37]. Nuclear factor 1 (NF1) family members are expressed in astrocytes [35]. The NF1A was one of the first transcription factors used as a marker of glial lineages, including oligodendrocytes and astrocytes, activity which is important for the maintenance of hippocampal astrocyte synapse-supporting functions [38]. NF1B and NF1X are also expressed in the adult brain olfactory bulb, hippocampus, cortex, and brainstem, where they co-localize with ALDH1L1, but were also observed in neurons [35]. 

### 2.3. Membrane Proteins

Astrocytes maintain ion, energy, and neurotransmission homeostasis by selectively moving molecules in and out of the cell via transmembrane channels and transporter proteins. Expression of many of them is highly specific for astrocytes and can be used to monitor their functional states. Glial connexins (Cx) form gap junction channels through which a glial network maintains homeostasis of the CNS. Cx26, Cx30, and Cx43 are the main astroglial hemichannel proteins. They provide exchange with the extracellular space, as well as channel-independent functions involving protein interactions, cell adhesion and intracellular signaling. Cx30 is expressed only in mature astrocytes and was found to be a critical regulator of synaptic strength by controlling its location of astroglial processes and modulating glutamate transport. Cx43 is a key player in brain development and is the most abundantly expressed also in the adult CNS [39,40]. Aquaporin 4 (AQP4) is responsible for water homeostasis throughout the brain. In astroglial cells, AQP4 is localized in the endfeet plasma membrane and is the most abundant water channel in the brain [41]. Interestingly, AQP4 is not present in oligodendrocytes, which makes it a good glial differentiating factor, but it is also present in ependymal cells and especially highly expressed in Bergmann glia [33]. Potassium inwardly-rectifying channels (KIR 4.1) are highly expressed in astrocyte processes [42]. They are responsible for controlling cell hyperpolarization during resting potential, and uptake of K^+^ from the synaptic cleft or its redistribution. In the normal adult CNS, KIR4.1 levels vary significantly in gray matter astroglia and its level is also downregulated in pathological conditions, but resulting K^+^ level changes may not be detectable [43]. Bestrophin 1 (BEST1) is a non-voltage-dependent transmembrane channel responsible for the calcium-dependent transport of chloride ions, permeable for GABA and glutamate. It is mainly expressed in astrocytes and in mice it was found distributed closer to the glutamatergic synapses than GABAergic synapses [44]. Two main types of glutamate Na^+^-dependent transporters are EAAT1 (GLAST) and EAAT2 (GLT1), abundant in fine astrocyte processes, but also variably expressed by all cell types in the CNS [45]. Besides the cell membrane localization, they co-compartmentalize with mitochondria, supplying glutamate as a source of fuel for the brain [46]. Because of disrupted glutamate and potassium signaling in neurological disorders, glutamate transporters are proposed to be engaged in their development. GABA transporters (GATs) are also widely distributed throughout the brain [47]. The most numerous in astrocytes is subtype 3 (GAT-3), localized mostly in processes that are adjacent to neuronal synapses [48,49]. GAT-1 is localized in distal astrocytic processes but in the majority is expressed in neurons [50]. 

### 2.4. Astrocyte Metabolic Markers

Since astrocytes are the key managers of energy metabolism in the brain, they have several functions specific to them. These are: glycogen synthesis and storage of energy reserve, fatty acid oxidation (FAO), cholesterol synthesis, gluconeogenesis, ketone bodies synthesis, lactate shuttle, and glutamate–glutamine shuttle along with ammonia utilization. Proteomic studies confirmed that among the astrocyte specific proteins those belonging to the metabolic pathways were the most enriched, including cellular ketone, organic acid, carboxylic, oxidation-reduction processes, and amino acid amine and small molecule catabolic processes [33]. 

Glucose is taken up by astrocytes from blood vessels by selective transporters GLUT1 [51]. Only astrocytes can store glucose, thanks to synthesis of glycogen with glycogen synthase (GS), and utilize it with glycogen phosphorylase (GP) [52]. The astrocyte cells from various brain regions contain heterogeneous levels of glycogen. During starvation, astrocytes are able to produce glucose in the brain by gluconeogenesis using glucose 6-phosphatase (G6PC) and fructose 1,6-bisphosphate (FBP). Those two enzymes catalyze the reactions opposite to glycolysis depending on the concentration of available substrates. In normal situations, astrocytes exhibit a higher glycolytic rate than neurons and robustly express the bifunctional enzyme 6-phosphofructo-2-kinase/fructose-2,6-bisphosphatase-3 (PFKFB3), converting fructose-6-phosphate to fructose-2,6-bisphosphate, which allosterically activates the rate-limiting glycolytic enzyme phospho-fructokinase (PFK), higher expressed in astrocytes than in neurons. Downstream in the glycolysis process, pyruvate is formed from phosphoenolpyruvate by the enzyme pyruvate kinase muscle isoform-2 (PKM2) [53]. Pyruvate can be transformed into oxaloacetate by a glial-specific anaplerotic enzyme, pyruvate carboxylase (PC), to feed the Krebs cycle [54]. It can be also incorporated into oxidative phosphorylation and its flux is carefully controlled by the activity of the enzyme pyruvate dehydrogenase (PDH), which in astrocytes is inactivated by pyruvate dehydrogenase kinase 4 (PDK4) phosphorylation preventing pyruvate conversion to acetyl-CoA [55]. Analysis of transcriptome demonstrated that PDK4 expression is 30 times higher in astrocytes than in neurons [56]. Pyruvate in astrocytes is shunted to the formation of lactate by the enzyme lactate dehydrogenase (LDH5 or LDHB). Lactate is an important energy substrate shuttled from astrocytes to feed neurons via monocarboxylate transporters (MCTs): MCT1 and MCT4 have much lower affinity (3.5 mM) for lactate than the neuronal MCT2 (<1 mM), thus shuttling it mostly towards neurons [57]. MCT1 and MCT4 are responsible also for pyruvate and ketone bodies transfer. MCT1 is expressed in astrocytes and oligodendrocytes but MCT4 only in astrocytes [58]. Both MCT1 and 4 are expressed also in Schwann cells [59].

FAO contributes approximately 20% of brain oxidative energy production in rat brains [60]. Gene set enrichment analyses of mouse transcriptomes revealed that genes encoding enzymes involved in FAO and amino acid catabolism are consistently more expressed in astrocytes than in neurons [61]. Fatty acids metabolism in neurons has never been proven. Therefore markers of long-chain fatty acid transport to mitochondria by citrate transporter proteins (CTP1a and CTP2) are selective to astrocytes. Acetyl-CoA from FAO in astrocytes is used for ketone bodies production, and astrocytes overexpress the limiting enzyme 3-hydroxy-3-methylglutaryl-CoA synthase 2 (HMGCS2), converting acetyl-CoA to acetoacetate.

Astrocytes are also responsible for synthesis of cholesterol needed for axonal growth and synaptogenesis. The SREBP cleavage-activating protein (SCAP) mediates cholesterol synthesis in astrocytes and oligodendrocytes, while 3-hydroxy-3-methylglutaryl-coenzyme, a reductase (HMGCR), catalyzes the rate-limiting step in cholesterol synthesis. Liver X receptor/retinoid X receptor (LXR/RXR) activates enhanced production of cholesterol and mediates transcription of its transport proteins, such as apolipoprotein E (APOE) and ATP-binding cassette transporter 7 (ABCA7) [62]. Astrocytic ApoE forms the lipoprotein complex with cholesterol and is secreted through the ABCAs [63]. Recent transcriptome analysis from human brain samples showed also expression of ApoE transcripts in microglia.

Astrocytes also overexpress enzymes related to the oxidation of unsaturated fatty acids, NADPH, Enoyl-CoA delta isomerase 1 and 2 (ECI1 and ECI2), and are responsible for membrane lipid metabolism and dynamics: 1-acylglycerol-3-phosphate O-acyltransferase 5 (ABHD5), glycerol kinase (GK), glycerol-3-phosphate acyltransferase (GPAT3) or isopentenyl-diphosphate delta-isomerase 1 (IDI1) [64]. 

Fatty acid-binding proteins (FABP) exhibit high affinity reversible binding of saturated and unsaturated long-chain fatty acids. FABP7 expression is observed in neural stem cells throughout development, while in adulthood it decreases and becomes restricted to radial glia-like cells and astrocytes [65]. In the adult brain, FABP7 is especially abundant in astrocytes that are rich in cytoplasmic granules that are believed to be originated from damaged mitochondria [66]. 

Astrocytes are enriched in expression of proteins responsible for catabolism of glycogenic amino acids such as glycine decarboxylase (GLDC), alanine aminotransferase 2 (GPT2), 3-hydroxyisobutyrate dehydrogenase (for valine) (HIBADH), serine hydrolase-like and serine dehydratase-like (SERHL and SDSL) or proline dehydrogenase 1 (PRODH) [64]. For example, L-serine is produced in astroglia from the glycolytic pathway via enzyme 3-phosphoglycerate dehydrogenase (3PGDH) enriched in glia and astrocytes during development [67].

Uptake and recycling of glutamate in the synapse also relies on astrocyte functioning. They selectively express glutamine synthetase (GLUL), enzyme converting glutamate to glutamine, and glutamine transporters that are particularly suited for glutamine release sodium-coupled neutral amino acid transporters (SNAT3 and SNAT5) [68]. 

A fraction of the synaptically-released glutamate is also likely to serve as an energy substrate by conversion of glutamate to alpha-ketoglutarate (AKG). Glutamate is transported to the mitochondria by the mitochondrial glutamate carrier 2 (GC-2) and oxidatively deaminated to AKG by glutamate dehydrogenase (GDH) [61]. GC-2 is predominantly expressed in protoplasmic astrocyte cells but also in oligodendrocyte progenitor cells. 

Glial cells are key players in the central control of energy balance and etiology of obesity. Interestingly, astrocytes express the glucagon-like peptide-1 receptor (GLP-1R), a modulator of food intake and body weight [69]. Astrocytes metabolize also thyroid hormones to actively form and incorporate circulating L-thyroxine (T4) through the solute carrier organic anion transporter family member 1C1 transporter (SLCO1C1, also known as OATP1C1) from blood. In astrocytes T4 is deiodinated by the type 2-deiodinase (DIO2) to produce 3, 3′5-triiodothyronine (T3). It has been shown that mitochondrial hydroxyacyl-CoA dehydrogenase/3-ketoacyl-CoA thiolase/enoyl-CoA hydratase alpha (HADHA) is critical for FAO regulation by T3. Since 95% of HADHA co-localizes with GFAP in the brain, T3 is considered to upregulate HADHA and subsequent neuroprotective mitochondrial energy production via FAO in astrocytes [70].

Care must be taken when using astrocyte enriched energy metabolism markers because some of them might be expressed also by microglial cells (for example PDK4), but there are often no specific studies clearly discriminating metabolism proteins specific to particular glial cell expression. Moreover, astrocyte activation involves profound changes in their energy metabolism (see below).

## 3. How Astrocytes Communicate with Other Cell Types

The instant communication between microglia, astrocytes, and neurons is required for immediate and adequate response to microenvironmental changes or external danger. One may say that neuroglia and neurons complement each other’s actions, and therefore their proficient communication is crucial for maintaining CNS environmental homeostasis. Studies showed that cytotoxic inflammation in astroglial cultures can occur exclusively in the presence of microglia, which express receptors recognizing pathogens and danger signals [71,72]. On the other hand, ongoing astrogliosis was restrained while microglia activation was suppressed [73,74]. This astrocyte-microglia interaction is essential in inflammation regulation. Astrocytes exchange signals with other glial cells, neurons, and blood vessels, which could be also used as marker molecules. These include signaling via gliotransmitters released in response to stimuli (i.e., neurotransmitters or intracellular Ca^2+^ levels increment) which may be divided into metabolic mediators (e.g., glutamate detected by NMDA receptors on neurons along with D-serine [75]) and tissue damage molecules (e.g., ATP [76]) [77]. Another group of factors engaged in cellular communication includes innate-immunity mediators such as cytokines, molecules examined in neuroinflammatory studies by default (i.e., interleukins (IL) such as IL-33 expressed by developing astrocytes [78], tumor necrosis factor (TNF) [79,80]), and chemokines) [81,82,83]. Extracellular vesicles (EVs) may be filled with active biomolecules, whose actions can influence the transcription profile in distinct cells. Experimental results showed that EVs contain some specific molecules, but a great part of their cargo is common [84]. Worth noting is that astroglial EVs have been called a two-edged sword due to their ability to transfer pathogenic factors in the course of neurodegenerative diseases, aside from their neuro-regenerative potential [85]. Astrocytes express the soluble NSF attachment protein receptor (SNARE), and its mechanism of action differs from that in neuronal activity (for review see [86]). Secreted glycoproteins (e.g., thrombospondins (TSPs)) are released postnatally by immature astrocytes in order to induce synaptogenesis in the developing brain, and stimulate cells to interact with proteases, cytokines, and growth factors or other cells via surface cell-adhesion receptors [87]). Growth factors’ release also serves as a cell-to-cell communication tool (e.g., for the growth-associated protein 43, GAP 43 [88]). Neurotrophins include polysialated neural cell adhesion molecules (PSA-NCAM) [89], ephrins [90], integrins [91], and different growth factors such as glial cell line-derived neurotrophic factor (GDNF) [92] or brain-derived neurotrophic factor (BDNF) [93]. Membrane channels, e.g., Cx, with prevalence described earlier for Cx30 and Cx43, are proteins forming gap junctions—free-flow channels connecting adjacent astroglial cells [94]. The ECM proteins secreted by astrocytes cannot be skipped in discussion about CNS cell communication. This composition of glycoproteins (including proteoglycans), glycosaminoglycans, heparin sulphate glycosaminoglycans (HS GAG), tenascin C (TN-C), TSP, secreted protein acidic and rich in cysteine (SPARC), and CYR61/CTGF/NOV (CCN) families [95] creates a dynamic environment for neurons and neuroglia. It provides structural support, trophic factors storage, allows short and long-distance communication between cells, and their migration and proliferation. Secretion of ECM factors, as well as enzymes modifying this environment such as matrix metalloproteinases (MMPs) or a disintegrin and metalloproteinase with thrombospondin motifs (ADAMTs), by glia can regulate neural plasticity and adaptive potential [96].

As described above, astrocytes can communicate with other CNS cells in various ways, which enables fluent signal transmission in physiological conditions in order to keep homeostatic balance. Some of those factors are specific enough for astrocytes to serve as markers of reactive astrogliosis.

## 4. Astroglia Activation

According to the current knowledge, the term activation is an oversimplification of a range of different phenotypes ‘activated’ in response to various physiological and pathological cell states, as it was published by Escartin et al. in 2021 [97]. Nevertheless, the activation of astrocytic cells causes their morphological change: somatic and dendritic hypertrophy, and processes elongation but not overall cellular volume change. These enhanced features along with cellular proliferation are called astrogliosis [97,98], which may be a consequence of injury, infection, or a direct activation by microglia. In this state astrocytes are also a source of inflammatory factors such as nitric oxide, prostaglandins, amino acids, or cytokines [99]. The direct astrocyte cooperation with microglia is very important in inflammation. Astrocytes are unable to migrate to the site of injury like microglia, but do proliferate and increase their number in the affected region [100]. 

Recently, Sofroniew (2020) suggested a preliminary division of activated astroglia into two subtypes. Based on their structure, proliferative state, the types of cells they interact with, and the tissue architecture to which they contribute he indicated: (i) astrocytes newly proliferated and organized into a new and permanent tissue architecture that forms borders around areas of inflamed tissue (called previously ‘glial scar’); and (ii) astrocytes that do not proliferate and retain the basic cell structure, tissue architecture, and functional interactions they exhibited in healthy tissue. Each of those subtypes can undergo a dynamic change in their biological state in time. Such changes in reactive astrocyte states may be finely graded and continuous, without a clear-cut division into a particular defined phenotype. The recognition of better defined particular astrocyte subtypes and their states requires more studies and focus on their markers [101].

### 4.1. What Activates Astrocytes, and What about A1/A2 Polarization?

The initiation of activation chain reaction is dependent predominantly on microglia cells, which express many more receptors recognizing pathogens than astrocytes or neurons. Astrocyte activation occurs later than rapid microglial; moreover, it is more prolonged in time [72,102,103,104]. Factors secreted by other CNS cells can induce astroglial activation, and those include cytokines (interleukins, interferons) [105,106], danger associated molecular patterns (DAMPs) [107,108], growth factors [109,110], hormones [111,112], neurodegeneration-associated proteins [113], and pathogen associated molecular patterns (PAMPs) [114,115,116].

The major breakthrough in astroglia polarization came with experiments investigating different stimuli activating particular astroglia phenotypes [29,117]. Astrocytic genes were profiled according to the way of stimulation: in LPS or ischemia induced activation, respectively, revealing huge phenotypic differences [29,71]. With reference to the microglia (and originally Th cells), astroglial activation phases were conventionally divided into classic A1 (inflammatory, cytotoxic), and alternative A2 (protective) [71,118]. Today it is known that A1 and A2 are two extremes in the polarization spectrum, and there exist also multiple intermediate and alternate phenotypes. More importantly, the black-and-white scenario of astrocyte population expressing only A1 or A2-classified markers in certain pathogenic conditions is not observed in vivo or even in vitro [117,118,119]. Unfortunately, current knowledge does not allow proposing definitive categorization in this matter yet [97,101]. A specific set of inflammatory factors, i.e., secreted by LPS-activated microglia (IL-1α, TNF, and C1q), was proven to trigger the activation of a cytotoxic phase in vitro to an extent comparable to that of direct LPS stimulation [71]. Those factors are reported to be necessary and sufficient to activate astrocytes. What is interesting is that excluding the same inflammatory factors cocktail from cellular surroundings does not reverse astrocytes’ activation. Astroglia polarized towards extreme A1 phenotype are considered as cytotoxic and may lead to cellular damage. This issue is raised often in the context of neurodegenerative diseases. On the other hand, the phenotype leaning towards A2 polarization is related to the promotion of synaptic formation, neurites’ growth, and anti-inflammatory factors’ production and release [120]. These are important observations in the context of future classification of astroglia polarization markers but clearly the subject is much broader than A1/A2 phenotype. 

### 4.2. Activated Astrocytes Markers

The great majority of reactive astrogliosis markers are not phenotype-specific but overexpressed, pan-reactive astrocytic proteins; therefore, they need to be examined in a quantitative way or using sets of multiple markers. Proteins considered as markers of astroglia reactivity may be divided into separate subgroups defined by optimal method for their detection: morphology-related (e.g., building cytoskeleton or transmembrane receptors), secreted (such as cytokines), and intracellular (e.g., chaperones, enzymes, transporters, or transcription factors) (Table 3).

### 4.3. Structural and Membrane Proteins

Among cytoskeleton-building proteins, GFAP is a commonly used marker of astroglial activation due to its strong increase in expression observed in most pathological conditions including neurodegeneration and injuries [121]. GFAP is not always a good choice for assessing the severity of a condition due to the variability in its basal levels between structures [122]. GFAP immunoreactivity is reflected by changes in the cytoskeleton, but its reorganization may not be followed by hypertrophy [98]. Other intermediate filaments whose expression increases after astrocyte activation are: vimentin, nestin, and synemin [123,124,125]. Changes in their expression levels differ in time-delay after activation and depend on the type of insult. Interestingly, nestin was not detected in the healthy adult cortex but following an innate immune challenge and after stroke its expression changes markedly, shifting the cellular expression patterns towards activated microglia/macrophages and astrocytes [29,124,126]. The six transmembrane epithelial antigen of prostate 4 (STEAP4) is a NADPH-dependent metalloreductase—a membrane protein reducing Fe^3+^ and Cu^2+^ ions. Its mRNA levels were found to be upregulated in inflammation, though this factor is not exclusive for astroglia [29,81]. Transmembrane proteins, such as Cx channels Cx30 and Cx43, were reported to be strongly engaged in inflammatory activation of astrocytes [127]. CD44 is a surface receptor for hyaluronan, responsible for cell interactions and communication with the environment and ECM. It is responsible for astroglia morphological changes and can be detected on long, unbranched human astrocytes [128,129,130]. Metabolic markers also contain proteins undergoing regulation of astroglial cells reactivity. The upregulation of FAO may be a common property of reactive astrocytes. It is consistent with the upregulation of the fatty acid transporter CD36 [131]. FABP7, also known as brain lipid-binding protein (BLBP), is a transporter expressed by injured astrocytes and a marker of Bergmann glia and radial glia [132,133]. In astrocytes from the spinal cord it induces a pro-inflammatory phenotype that renders these cells toxic to motor neurons in coculture [134].

The following proteins are considered as engaged in the protective astrocyte activation spectrum. Sphingosine-1 phosphate receptor 3 (S1P3) is a cell-membrane protein overexpressed by astrocytes in the course of neuroinflammation as it was confirmed in rodents and human patients. It has the ability to modulate blood–brain and blood–tumor barriers’ permeability [135]. Transglutaminase 1 (TGM1), a membrane-bound enzyme engaged in cornified cell envelopes (layer of highly insoluble proteins deposited on the inner surface of the plasma membrane), acts as a mechanical barrier against infectious agents [136]. Epithelial membrane protein (EMP1) is a small membrane glycoprotein regulating, among other things, cellular proliferation [137]. A cluster of differentiation receptors also undergo upregulation on A2 activated astroglia, including CD109 and CD14 receptors [29,71,138]. 

### 4.4. Secreted Proteins

Lipocalin 2 (LCN2 or NGAL) is a secreted protein whose role is to bind iron ions. It participates in innate immunity and apoptotic mechanisms with strong upregulation during reactive astrogliosis, but may be expressed also by microglia and endothelial cells [139]. Together with serine protease inhibitor A3N (Serpin A3N), a secreted peptidase inhibitor, they are induced in activated astrocytes, while their expression in resting state cells is below detection level. The Serpin A3N gene was found to be upregulated only in astrocytes. Both LCN2 and Serpin A3N colocalize with the GLAST transporter [29,140,141]. Thrombospondin-1 (TSP-1) is a signal transducer and activator of transcription 3 (STAT-3)-regulated factor stimulating synaptogenesis and was found to be upregulated after motor neurons’ injury along with GFAP [142]. Cortical metallothionein (MT), a metal-binding protein acting as an antioxidant, was found overexpressed in neurodegenerative diseases [138]. TN-C is a matricellular protein. Aside from its engagement in astroglia development and synaptic plasticity, it is a marker of reactive astrocytes in stroke, neuronal injury, and glial scar formation [95,140,143]. A tissue inhibitor of metalloproteinase 1 (TIMP1) forms complexes with MMPs by binding to their catalytic zinc cofactor, which results in irreversible inactivation. It is able to induce reactive astrogliosis, and is necessary to its maintenance in IL-1β-stimulated cell culture [144]. It is also produced by astrocytes in response to pathophysiological threats [29]. The milk fat globulin protein E8 (MFG-E8) is a secreted protein, which is indirectly responsible for removal of damaged neurons [145,146]. C-X-C motif chemokine 10 (CXCL10) is a pro-inflammatory protein acting on the CXCR3 receptor. It mediates an inflammatory response between neurons and glia [138].

Pathological conditions leading to pro-inflammatory reactive astrogliosis may cause release of certain proteins by astrocytes. The first line of response is rapid expression of cytokines (e.g., IL-1β, IL-6, TNF-α), IFN-γ, and chemokines (e.g., CCL2, CCL5, CXCL) [118,147,148,149]. Complement component 3 (C3) is known to be upregulated in the course of neurodegenerative diseases, but is also expressed by microglial cells [71,150]. Complement factor B (CFB), similar to C3, is also an element of alternate complement pathway activation which modulates proliferation and degradation of blood cellular components during inflammation [71,151]. Plasma protease C1 inhibitor (Serpin G1) interferes with C3 and CFB by physical binding, influencing the alternate complement activation [152]. The interferon-induced GTP-binding protein (MX1S) acts against viral nucleic acids [71,153]. S100β can be actively secreted from astrocytes during cellular stress along with downregulation of its expression intracellularly [154]. Moreover, upregulated levels of this protein detectable in serum of patients after brain injuries correlate with the severity of the insult [155].

Among factors released from activated astrocytes with putative contribution to cytoprotection, cytokines open the list (IL-1Ra, IL-10, TGFβ) [150]. A chemokine-like protein, prokineticin-2 (PK2), stimulates and promotes shift of the astroglia phenotype into immunosuppressive when overexpressed by neurons and astrocytes [118]. Pentraxin 3 (PTX3) is directly related to the innate immunity response to pathogens. After secretion it activates the classical complement pathway. It is also responsible for astroglial support of BBB integrity after ischemic stroke [118,156]. 

### 4.5. Intracellular Proteins

Fructose-bisphosphate aldolase C (ALDOC) is a glycolytic enzyme found to be upregulated after spinal or brain injuries [157]. It has been found in CSF of patients after traumatic brain injury where ALDOC along with FABP7 and PEA15 are expressed by astrocytes [158]. Increased expression of the monoamine oxidase B (MAO-B) enzyme is a characteristic marker in neurodegenerative conditions affecting neurons releasing monoamines [159]. Lipid transporters are also upregulated in pathological conditions involving astrogliosis. Translocator protein (TSPO) is a mitochondrial transporter used in PET imaging, but is expressed also by microglia and vascular cells [160]. Chaperones HSPB5 (also known as CRYAB) and HSPB1/HSP27 are overexpressed in neurodegenerative diseases and these heat shock proteins are conservative among different species [161]. Among transcription factors, the nuclear factor of activated T cells’ (NFAT) reaction is sensitive to Ca^++^ signaling, and tropomyosin receptor kinase B (TrkB) and IL-17 receptor (IL-17R) engage BDNF signaling and the NFκB pathway with NO production [162,163]. SRY-box transcription factor 9 (SOX9) is found to be almost exclusively expressed by astrocytes in the adult human CNS, and its strong upregulation was reported in a rodent amyotrophic lateral sclerosis model [34]. The STAT3, JAK-STAT pathway element is widely expressed by many cell types but in astrocytes is responsible for processes formation and GFAP distribution. It was reported to be necessary for astroglial differentiation [164] and is also overexpressed by astroglia in injury-induced inflammation [142,165]. S100β, a Ca^++^-binding protein, has been reported to develop features of Parkinson’s disease (PD), such as the impairment of motor coordination and the expression of some molecular parameters, including the dopamine D2 receptor [166]. The S100A10-coding gene (S100a10) was reported to be upregulated in neurodegenerative-like conditions. Its physiological function is to modulate cell proliferation, membrane repair, and inhibition of cell apoptosis [127,167].

Cytoplasmic inducible NO synthetase (iNOS, or NOS2), an enzyme engaged in cytokine-induced NO production during the inflammatory response, was reported to be strongly upregulated not only in microglia but also in reactive astrocytes [118]. Guanylate-binding protein 2 (GBP2) enzyme reduces GTP by hydrolyzation, which during inflammation has anti-viral properties [118]. N-Myc downstream-regulated gene 2 (NDRG2) is a tumor suppressor and cellular stress-related protein associated with cell proliferation and differentiation. Importantly, it is exclusively expressed by astrocytes. Its cytoplasmic localization allows visualization in cell bodies and processes. Its involvement in pathogenesis development was reported in experimental autoimmune encephalomyelitis mice and ischemia models; however, it may act through a different signaling pathway causing opposite effects [168,169,170].

Astrocytic transcription regulating nuclear factor erythroid 2-related factor 2 (NRF2) was reported to act against neurotoxic consequences in dopaminergic (DA) neurons when overexpressed [118]. Its physiological role is to regulate gene expression related to the oxidative stress and redox reactions [171]. NRF2 is expressed exclusively in astrocytes in the course of PD, but additionally in microglia and neurons in Alzheimer’s disease (AD). The expression pattern of this factor appears to be dependent on complex conditions [172]. Arginase 1 (ARG1) is an enzyme converting L-arginine in the urea cycle. Arginine metabolism is crucial in immune cells responses, engaging polymorphonuclear granulocytes and further T cells and NK cells [118]. Along with microglia its expression was also found in astrocytes. Moreover, its putative neuroprotective impact has been described in amyloid beta-related neurodegeneration [173]. The sphingosine kinase-1 (SPHK1) enzyme catalyzes the phosphorylation of sphingosine, which leads to the activation of NFκB signaling and IL-17 secretion [174]. Intracellular prostaglandin G/H synthase 2 (PTGS2) during inflammation is responsible for stimulation of prostaglandins production. The level of expression of both these factors was increased in a middle cerebral artery occlusion (MCAO) stroke model in mouse and inflammation-related reactive astrogliosis [29].

## 5. Heterogeneity of Astrocytes

Astrocytes are present in all brain areas, but their density and neuron-glia ratio are not the same throughout the tissue. For example, subcortical brain regions have neuron-glia ratio of approximately 11:1 while in the cortex it is 4:1 [175]. The density of astroglia ranges from 2–3 cells/mm2 in the core of the nucleus accumbens to about 2500 cells/mm2 in the subventricular zone [3]. Importantly, regional astroglial morphology, density, and proliferation rates vary significantly when measured using different markers [3]. Astrocytes are the most numerous glial cells in the CNS (see Clarke et al. 2020) [36] and the term astroglia refers to a plethora of heterogeneous types, depending on their morphology, localization, function, or often determined by the type of neurons they assist. It is important to keep that in mind in biochemical experiments’ design, due to the strong recommendations regarding markers used for astroglia visualization, because they may express different levels of basic markers such as GFAP, and using only this one determinant in assessing their proliferation or number may lead to false conclusions [3,97]. The most robust astroglial subpopulations are described below.

Protoplasmic astrocytes are predominant astroglial cells in the gray matter. They are intensively ramified, which enables effective connection with neighboring neurons and blood vessels [176]. They have relatively low expression of GFAP. Fibrous astrocytes, on the other hand, are localized in white matter. They possess long processes, with compact distribution but numerous extensions. Those cells contact synaptic terminals in subpial region, as well as axons at nodes of Ranvier. Fibrous astroglia expose high expression of GFAP and have rather a structural function. Radial astrocytes are bipolar cells with oval soma and long processes. One of them is localized near the ventricular wall and the other at the pial surface. They are numerously present in the developing embryonic brain, where they form scaffolding for migrating neurons, and support neuroblast survival by expressing, for example, epidermal growth factor (EGF) or glial cell-derived neurotrophic factor (GDNF). In the adult brain they remain in the retina as Mūller cells. Bergmann glia present in the cerebellum contact multiple (even several thousand each) synapses of Purkinje neurons thanks to their extensive branching. They are enriched in AMPA receptors (GluA, GluA4, GLAST) and sonic hedgehog (SHH) signaling pathway proteins (GLI1 transcription factor and PTCH1/2 receptors). In contrast, these genes’ expression is low in velate astrocytes, which are also localized in the cerebellum and express large amounts of the water channel AQP4. These cells have thick, short, and extensive processes, which serve as protection for granule neurons in a 1:1 ratio. Varicose projection astrocytes and interlaminar astrocytes are found only in primates [4,19]. Additionally to that described above, other astroglial cell types can also be distinguished, such as perivascular and marginal astroglia, tanycytes, epandymocytes, choroid plexus cells, retinal pigment epithelial cells, and pecten glial cells [177].

Particular types of astrocytes can be found in specific brain structures. For example, the amygdala has predominantly fibrous and perivascular astrocytes, the hypothalamus contains mostly protoplasmic astroglia, while in the hippocampus there are protoplasmic, radial, and velate astrocytes. The olfactory bulb contains also protoplasmic astrocytes in the condensed gray matter, while Bergmann glia is present in the cerebellum [178,179]. 

Not only do astrocytes located in the remote brain regions have distinct markers, but they are also heterogenous within the same structure [3]. Differentiation can be provided based on transcriptional profiles [180]. Notably, single-cell RNA sequencing methods are now gaining plenty of precise information in relation to this aspect [181,182]. In the cortex there are morphological differences between astrocytes located in its particular layers. These are related to the differences in neuronal migration during development [180]. Cortical astrocytes express low levels of GFAP and are mostly located in the first and deep layers and subcortical regions [18]. GAT-3 and Kir4.1 expression prevails in layers 2/3 and 5, whereas AQP4, GFAP, and Cx43 prevail in layer 1 and in pial astrocytes [183,184]. Striatal astrocytes, compared to the hippocampal, express aldehyde dehydrogenase 5 family member A1 (ALDH5A1), a protein involved in GABA degradation. On the other hand, hippocampal astrocytes compared with the striatal have higher expression of GFAP, Cx43, and GS [185]. The ventral tegmental area (VTA) has more astrocytes than the substantia nigra (SN) region, which might be the reason for enhanced neuronal resistance to damaging factors when comparing these two structures under pathological conditions, e.g., in PD [186]. Expression of growth factor GDF15 (a member of the TGFβ superfamily, known also as macrophage inhibitory cytokine 1) in the VTA is 230-fold higher than that in the SN [187]. Astrocytes in these two structures have low expression of Kir4.1 channels [188]. Astrocytes in VTA express higher amounts of interleukins (IL-10) and chemokines (CCL3, 6, 12, and CXCL13). Hypothalamus and thalamus astrocyte RNA-Seq datasets are in the most cases different from other regions [1]. Dorsal Bergmann glia in the cerebellum express the majority of EAAT1/GLAST transporter [51,189,190]. Two morphologically different GFAP-positive cell populations were described in the hippocampus: one expressing GLT1 and the other GLAST [191]. The differences were also described in the spinal cord, where Reelin, Slit1, and Nkx6.1 and Pax6 transcription factors, were diversely expressed depending on the domain localization [192]. 

## 6. Astroglial Markers in the Aging Brain

The presence of particular astrocyte types and their markers changes throughout the life span, but there is no decrease in general astrocyte numbers related to aging [193,194,195]. Aging affects their physiology and is often implicated in neurological diseases; therefore, astrocytic markers are important for diagnostic use. What is interesting is that the physiological aging triggers astrocyte activation, which may contribute to lowered antioxidative potential, loss of plasticity in formation, and elimination of synapses. Aged astrocytes become pro-inflammatory, with enhanced expression of activation markers, especially in the hippocampus and corpus striatum [196]. The main phenotypic changes of astrocytes promote generation of an environment favoring neuronal damage in senescent organisms, which is also observed in preclinical studies on mice [47,197]. Moreover, astrocytes tend to change their localization-dependent set of molecular markers with age, unlike other glial cells and neurons, which was reported in AD and PD human patients, as well as in rodents [47,193,196]. Senescent astrocytes from post mortem human tissue showed enhanced expression of inflammatory markers, potentially toxic to DA neurons; therefore, aging cells were proposed to become a tool for studying PD-related neurodegeneration [197].

The majority of markers which show enhanced expression during aging are those with a specific cytotoxic phenotype activation pattern. Several CNS pathophysiological conditions were examined by Liddelow et al. and revealed that GFAP/C3-positive astrocytes were present in PD patients’ SN, AD prefrontal cortex and hippocampus, amyotrophic lateral sclerosis (ALS) motor cortex, Huntington’s disease (HD) caudate nucleus, and multiple sclerosis (MS) subcortical tissue [71]. The astroglial role in neurodegeneration is perceived as a two-edged sword. Astrocytes could participate in pathology of multiple CNS diseases due to the loss of their protection and/or due to the toxic gain of function. 

PD symptoms manifest when advanced pathology is already in progress. Glia contribution to the pre-symptomatic neurodegeneration is now extensively studied. Overexpressed glia-characteristic protein markers, such as GFAP, GMBF, galectin 1, and sorcin A, originally linked with an astroglial putative cytoprotective response towards remaining neurons, were found in the SN [198]. Greater levels of the neuroprotective protein GDF15, expressed originally in astrocytes, were found in the cerebrospinal fluid of PD patients who showed later disease onset [199]. Reports based on GFAP, vimentin, MT I and II, and HSP27 immunolabeling showed a lack of actual reactive astrogliosis in human post mortem tissue of PD-affected SN brain regions [200,201]. However, protein aggregates can be also found in few astrocytes and astrocyte-related energy metabolism changes were described [202].

Examination of post mortem tissue samples from patients suffering from AD revealed that about 60% of GFAP-positive astrocytes in the prefrontal cortex were also C3-positive [119,203]. Interestingly, astroglia analysis from AD patients’ tissue without dementia showed high expression of the GLT1 transporter, which suggests the presence of reactive astrocytes expressing A2 spectrum range markers [204]. Though it was demonstrated that EAAT2 is strongly upregulated in the course of AD in patients [204], there are also animal experiments where their levels remain unchanged [205], so the issue of their contribution remains open. Reactive astrocytes are shown to be present in amyloid beta senile plaques and neurofibrillary tangles [206]. This probably may contribute to altered homeostasis and cognitive impairment, and in further stages to neurite plaque formation [207]. Although APOE is a protein expressed by non-neuronal cells, it was also found upregulated in neurons in AD brains, as a pathology-related occurrence [161].

HD development is related to mutation of the huntingtin-coding gene (HTT). Mutant huntingtin (mHTT) was originally studied in neurons, and now it is known that astrocytes also express it, which drives reactive astrogliosis development [208]. Severity of this activation is related with disease progression (from mild in grade 0 HD to severe in grade 4, respectively in ascending order) as measured in an animal transgenic model by evaluation of GFAP expression in the striatum [209]. Screening experiments revealed genes upregulated in HD patients’ tissue: GFAP, CD44, OSMR, FKBP5, STEAP4, and CXCL10 (pan-reactive); C3, SRGN, and GBP2 (A1-reactive); EMP1, CD14, and CD109 (A2-reactive) [138]. Consequent dysfunction of striatal and cortical neurons leads to clinical symptoms’ development. 

ALS involves degeneration of motor neurons localized in the cortex, brainstem, and spinal cord. Reactive astrocytes in this condition have been found in later phases of disease and neurodegeneration in mice models, and while activated they became neurotoxic [210]. The inflammatory response is proposed to be engaged in development of ALS, due to reported overexpression of astroglial (and microglial) CD44 glycoprotein in mice spinal cord tissue [130]. Human embryonic stem cells-derived astroglia were used in a murine ALS model. Cells were implanted intrathecally and kept their physiological functions without unwanted cytotoxicity and proliferation [211].

There is a significant number of reports showing that stroke (ischemia) induces reactive astrogliosis through distinct mechanisms other than neuroinflammation (which may be induced, e.g., by LPS). There is, however, a set of changes in gene expression common for the two types of activation which was revealed after rat brain transcriptome analysis [9,29]. Astrocytes expressing neuroprotective markers able to stop ongoing inflammation processes have been found in animal models of ischemic injuries and are able to heal wounds in astroglial cultures [71,118].

Reactive astrogliosis is often a marker of neuronal damage. Astrocytes maintain the homeostasis of surrounding tissue, govern energy metabolism, and give protection and trophic care, modulating plasticity and inflammation as well. Therefore, there are very few factors that could cause CNS pathology without astroglial direct or indirect involvement, making astrocytic proteins excellent candidates for disease diagnostic markers. 

## 7. Perspectives

Looking forward into the future of neurodegeneration-related scientific research, it is important to appreciate astrocytes as active neuronal partners, and to remember that non-neuronal cells also take part in the pathological process. They may be affected, but also may affect other cells. In order to clarify this issue, the heterogeneity of astroglia has to be kept in mind, and studies should be adjusted to narrow cell subtype in their exact spatial and temporal phenotype. Astrocytes are dynamic, functionally flexible, and express multiple states depending on their neighborhood and situation. They promote both protective and toxic outcomes, so it is worth noting that functional studies would be more informative than those focused merely on morphological changes. At best, multiple protein markers are advised to be used for research on differential astrocyte states. This review is focused on astroglia but it has to be kept in mind that the glia family consists of other cells, which along with neurons are in constant communication with each other (for additional information see review [72]).

## Figures and Tables

**Table 1:** List of protein markers engaged in functions and processes specific to astrocytes.

Specific Astrocytic Function	Marker Protein
**Glucose uptake**	GLUT1
**Gluconeogenesis**	G6PC, FBP, PC
**Glycogen synthesis and use**	GS, GP
**Glycolytic metabolism**	PFKFB3, PFK, PKM2, PC, PDK4
**Lactate shuttle**	LDH5, MCT4
**Ketone body synthesis**	HMGCS2
**Fatty acid oxidation and transport**	CPT1a, CPT2, FABP7
	ECI1, ECI2, ABHD5, GK, GPAT3, IDI1
**Cholesterol synthesis**	SCAP, HMGCR, LXR/RXR, APOE, ABCA7
**Catabolism of glycogenic amino acids**	GLDC, GPT2, HIBADH, SERHL, SDSL, PRODH, 3PGDH
**Glutamate–glutamine shuttle**	GLUL, SNAT3, SNAT5
**Excitatory amino-acid transporters**	EAAT1, EAAT2
**Water transport**	AQP4

**Table 2:** Characterization of general astrocyte protein markers.

Protein Marker	Full Name	Information
**Structural and Membrane Proteins**		
AQP4	aquaporin 4	water homeostasis throughout the brain; localized in endfeet plasma membrane; highly expressed on Bergmann glia; not present at oligodendrocytes
BEST1	bestrophin 1	calcium-dependent transport of chloride ions, permeable for GABA and glutamate; mainly expressed in astrocytes; in mice distributed closer to the glutamatergic synapses than GABAergic synapses
CNN1	calponin-1	actin-related processes; enriched in astrocytes
Cx26	connexin 26	forms gap junction channels in glial network to maintain homeostasis of the CNS
Cx30	connexin 30	critical regulator of synaptic strength, controls location of astroglial processes and modulates glutamate transport; expressed only in mature astrocytes, mostly gray matter
Cx43	connexin 43	key player in brain development; abundantly expressed in adult CNS astrocyte endfeet
EAAT1 (GLAST)	excitatory amino acid transporter 1, glutamate aspartate transporter	glutamate uptake, co-compartmentalizes with mitochondria, supplying glutamate as a fuel for the brain; abundant in fine astrocyte processes, but also variably expressed by all cell types in the CNS
EAAT2 (GLT1)	excitatory amino acid transporter 2; glutamate transporter-1	glutamate uptake, co-compartmentalizes with mitochondria, supplying glutamate as a fuel for the brain; abundant in fine astrocyte processes, but also variably expressed by all cell types in the CNS
ezrin	ezrin	protein anchoring the plasma membrane to cytoskeletal microfilaments; acts as stabilizer of filopodia (peripheral astrocyte processes)
FMN2	formin-2	actin-related processes; enriched in astrocytes
GAT-1	sodium- and chloride-dependent GABA transporter 1	GABA transporter; localized in distal astrocytic processes but in majority is expressed in neurons
GAT-3	sodium- and chloride-dependent GABA transporter 3	GABA transporter; localized mostly in processes adjacent to neuronal synapses; most numerous subtype in astrocytes
GFAP	glial fibrillary acidic protein	contributes to astroglial BBB mechanical support; expressed mainly by white matter astrocyte extensive branching and slightly their cell body; useful marker for studying complex astroglial morphology
KIR4. 1	potassium inwardly-rectifying channels	controls cell hyperpolarization during resting potential, uptake of K^+^ from the synaptic cleft or its redistribution; in the normal adult CNS, KIR4.1 levels vary significantly in gray matter astroglia, downregulated in pathological conditions
NEBL	LIM zinc-binding domain-containing Nebulette	actin-related processes; enriched in astrocytes
nestin	nestin	marker of stem or progenitor cells
PDLIM7	PDZ and LIM domain protein 7	actin-related processes; enriched in astrocytes
radixin	radixin	connect the plasma membrane with cytoskeletal microfilaments; act as stabilizer of filopodia (peripheral astrocyte processes)
RSPH1	radial spoke head 1 homolog	actin-related processes; astrocyte specific
synemin	synemin	intermediate filament; co-expressed by immature astrocytes along with GFAP, vimentin and nestin
SYNPO2	synaptopodin-2	actin-related processes; enriched in astrocytes
transitin	transitin	intermediate filament; expressed by radial glia during CNS development
vimentin	vimentin	role in differentiation and maturation of astrocytes; mostly expressed in some specialized glial cells such as Bergmann and radial glia or ependymal cells, white matter astrocytes
**Transcription Factors and Intracellular Proteins**		
NF1A	nuclear factor 1	maintains hippocampal astrocyte synapse-supporting functions; marker of glial lineages, including oligodendrocytes and astrocytes
NF1B	nuclear factor 2	expressed in the adult brain olfactory bulb, hippocampus, cortex, and brainstem where they co-localize with ALDH1L1, also observed in neurons
NF1X	nuclear factor 3	expressed in the adult brain olfactory bulb, hippocampus, cortex, and brainstem where they co-localize with ALDH1L1, also observed in neurons
NKX2-1	NK homeobox gene-coded protein	regulates GFAP expression; controls astroglia production spatiotemporally in embryos
NKX3-1	NK homeobox gene-coded protein	astrocytic region-specific transcription factor; exclusively expressed in astrocytes in olfactory bulb
NKX6-1	NK homeobox gene-coded protein	astrocytic region-specific transcription factor; exclusively expressed in astrocytes in brainstem
SOX9	SRY-related high mobility group (HMG) box gene 9	nuclear marker; expressed almost exclusively in astrocytes; good astroglia marker in adult brain, outside the neurogenic zones, upregulated in reactive astrocytes
S100β	S100 calcium-binding protein β	cytosolic Ca^++^-binding protein, also secreted; labels cell bodies of small astrocytes with less extended branchinga, also expressed in neonatal oligodendrocytes, adult NG2 glia, ependymocytes and spinal, medullar, pontine and cerebellar neurons
**Metabolic Markers**		
**Glucose Related**		
ALDH1L1	folate enzyme aldehyde dehydrogenase 1 family member L1	converting NADP to NADPH; not homogenous expression, expressed in fibrous and protoplasmic astrocytes, and radial glia
FBP	fructose 1,6-bisphosphate	gluconeogenesis; production of glucose during starvation, astrocyte specific
G6PC	glucose 6-phosphatase	gluconeogenesis; production of glucose during starvation, astrocyte specific
GLUT1	glucose transporter	glucose transport; less glycosylated isoform (45 kDa) found in astrocytes, more heavily glycolsylated isoform (55 kDa) is predominant in microvascular endothelium
GP	glycogen phosphorylase	glycogen utilization; astroglia-specific enzyme, degrades glycogen deposits in astroglia
GS	glycogen synthase	glycogen synthesis; selective to astrocytes, glycogen storage
LDH5	lactate dehydrogenase	conversion of pyruvate to lactate; bidirectional enzyme biased towards the production of lactate, twofold enriched in astrocytes
MCT1	monocarboxylate transporter 1	responsible for pyruvate, lactate and ketone bodies transfer; expressed in astrocytes and oligodendrocytes lower affinity for lactate than the neuronal MCT2, expression is age-dependent
MCT4	monocarboxylate transporter 4	responsible for pyruvate, lactate and ketone bodies transfer; expressed only in astrocytes, much lower affinity for lactate than the neuronal MCT2, low affinity for ketone bodies
PC	pyruvate carboxylase	converts pyruvate into oxaloacetate in glycolysis; glial-specific anaplerotic enzyme, providing oxaloacetate for the TCA cycle
PDK4	pyruvate dehydrogenase kinase 4	phosphorylation-mediated inactivation of PDH; 30 times higher expression in astrocytes than in neurons; expressed also by microglial cells
PFKFB3	6-phosphofructo-2-kinase/fructose-2,6-bisphosphatase-3	converts fructose-6-phosphate to fructose-2,6-bisphosphate, regulates glycolysis and gluconeogenesis; enriched in astrocytes, a magnitude lower expression in neurons due to continuous degradation
PFK	phospho-fructokinase	higher expressed in astrocytes than in neurons
PKM2	pyruvate kinase muscle isoform-2	converts phosphoenolpyruvate to pyruvate, regulates glycolysis; enriched in astrocytes, expressed also by other glial cells
**FAO Related**		
ABCA7	ATP-binding cassette transporter 7	cholesterol transporter
ABHD5	1-acylglycerol-3-phosphate O-acyltransferase 5	membrane lipid metabolism and dynamics
APOE	apolipoprotein E	forms lipoprotein complex with cholesterol and is secreted through the ABCAs; expressed mainly in astrocytes but also found in microglia, found in neurons under pathological conditions
CTP1a	carnitine palmitoyltransferase 1a	citrate transporter protein, essential step for the mitochondrial uptake of long-chain fatty acids and beta-oxidation in mitochondrion; selective to astrocytes in outer membrane of mitochondrion, rate-limiting step of beta-oxidation
CTP2	carnitine palmitoyltransferase 2	citrate transporter protein, essential step for the mitochondrial uptake of long-chain fatty acids and beta-oxidation in mitochondrion; localized in mitochondrion inner membrane
DIO2	type 2-deiodinase	removes iodide from L-thyroxine to produce 3, 3′5-triiodothyronine; thyroid hormone metabolism
ECL1	enoyl-CoA delta isomerase 1	oxidation of unsaturated fatty acids
ECL2	enoyl-CoA delta isomerase 2	oxidation of unsaturated fatty acids
FABP7	fatty acid-binding protein 7	high affinity reversible binding of saturated and unsaturated long-chain fatty acids; expression in neural stem cells throughout development, in adulthood decreases and becomes restricted to radial glia-like cells and astrocytes, abundant in astrocytes rich in cytoplasmic granules
GC-2	mitochondrial glutamate carrier 2	glutamate transport to the mitochondria; predominantly expressed in protoplasmic astrocyte cells but also in oligodendrocyte progenitor cells
GDH	glutamate dehydrogenase	oxidative deamination of glutamate to alpha-ketoglutarate
GK	glycerol kinase	membrane lipid metabolism and dynamics
GLDC	glycine decarboxylase	catabolism of glycogenic amino acids
GLP-1R	glucagon-like peptide-1 receptor	modulator of food intake and body weight
GLUL	glutamine synthetase	converts glutamate to glutamine; selectively expressed by astrocytes
GPAT3	glycerol-3-phosphate acyltransferase	membrane lipid metabolism and dynamics
GPT2	alanine aminotransferase 2	catabolism of glycogenic amino acids
HADHA	hydroxyacyl-CoA dehydrogenase/3-ketoacyl-CoA thiolase/enoyl-CoA hydratase alpha	critical for the FAO regulation by T3; 95% of HADHA co-localize with GFAP in the brain
HIBADH	3-hydroxyisobutyrate dehydrogenase	catabolism of glycogenic amino acids
HMGCR	3-hydroxy-3-methylglutaryl-coenzyme A reductase	catalyzes the rate-limiting step in cholesterol synthesis
HMGCS2	3-hydroxy-3-methylglutaryl-CoA synthase 2	converts acetyl-CoA to acetoacetate; rate-limiting enzyme in ketone bodies production, astrocyte specific
IDI1	isopentenyl-diphosphate delta-isomerase 1	membrane lipid metabolism and dynamics
LXR/RXR	liver X receptor/retinoid X receptor	activates production of cholesterol and mediates transcription of its transport proteins APOE and ABCA7
PRODH	proline dehedrogenase 1	catabolism of glycogenic amino acids
SCAP	SREBP cleavage-activating protein	mediates cholesterol synthesis; expressed in astrocytes and oligodendrocytes
SDSL	serine dehydratase-like	catabolism of glycogenic amino acids
SERHL	serine hydrolase-like	catabolism of glycogenic amino acids
SLCO1C1	solute carrier organic anion transporter family member 1C1 transporter	transports L-thyroxine from the blood; thyroid hormone metabolism
SNAT3	sodium-coupled neutral amino acid transporter 3	glutamine transporter; suited for glutamine release
SNAT5	sodium-coupled neutral amino acid transporter 5	glutamine transporter; suited for glutamine release

**Table 3:** Characterization of protein markers of reactive astrogliosis divided by cellular localization.

Protein Marker	Full Name	Information
**Structural and Membrane Proteins**		
CD109	cluster of differentiation receptor 109	upregulated in pro-inflammatory astroglial activation, specific for astrocytes, low expression after LPS, high after MCAO
CD14	cluster of differentiation receptor 14	upregulated in pro-inflammatory astroglial activation
CD36	cluster of differentiation receptor 36	fatty acid transporter; upregulated in reactive astrocytes
CD44	cluster of differentiation receptor 44	surface receptor for hyaluronan; responsible for astroglia morphological changes; detected on long, unbranched human astrocytes
Cx30	connexin 30	transmembrane protein; strongly engaged in inflammatory activation of astrocytes
Cx43	connexin 43	transmembrane protein channel; engaged in inflammatory reactive astrogliosis
EMP1	epithelial membrane protein	small membrane glycoprotein; regulates cellular proliferation
FABP7/BLBP	fatty acid-binding protein/brain lipid-binding protein	expressed in Bergmann and radial glia, and also by injured astrocytes
GFAP	glial fibrillary acidic protein	cytoskeleton-building protein; strong increase in expression observed in most pathological conditions including neurodegeneration and injuries; variability in its basal levels between structures
nestin	nestin	intermediate filament; expression increases after astrocyte activation by MCAO or stroke but not LPS
S1P3	sphingosine-1 phosphate receptor 3	cell-membrane protein; overexpressed by astrocytes in neuroinflammation, confirmed in rodents and humans; ability to modulate BBB permeability, activated by MCAO
STEAP4	six transmembrane epithelial antigen of prostate 4	membrane protein reducing Fe^3+^ and Cu^++^ ions; upregulated in inflammation, although not exclusive for astroglia
synemin	synemin	intermediate filament; expression increases after astrocyte activation
TGM1	transglutaminase 1	membrane-bound enzyme; creates mechanical barrier against infectious agents, specific for astrocytes, low expression after LPS, high after MCAO
vimentin	vimentin	intermediate filament; expression increases after astrocyte activation, both after MCAO and LPS
**Secreted Proteins**		
C3	complement component 3	upregulated in course of neurodegenerative diseases, also expressed by microglia
CFB	complement factor B	element of alternate complement pathway activation modulating proliferation and degradation of blood cellular components during inflammation
CXCL10	C-X-C motif chemokine 10	pro-inflammatory protein; mediates an inflammatory response between neurons and glia
LCN2	lipocalin 2	secreted protein-binding iron ions; participates in innate immunity and apoptotic mechanisms, strong upregulation during reactive astrogliosis, expressed also by endothelial cells and microglia; colocalizes with GLAST
MFG-E8	milk fat globulin protein E8	secreted protein; responsible for removal of damaged neurons
MT	metallothionein	Metal-binding protein; found overexpressed in neurodegenerative diseases
MX1S	interferon-induced GTP-binding protein	acts against viral nucleic acids
PK2	prokineticin-2	chemokine-like protein; stimulates and promotes shift of astroglia phenotype into immunosuppressive when overexpressed by neurons and astrocytes
PTX3	pentraxin 3	directly related to the innate immunity response to pathogens; responsible for astroglial support of BBB integrity after ischemic stroke; activates classical complement pathway, specific for astrocytes, low expression after LPS, high after MCAO
Serpin A3N	serine protease inhibitor A3N	expression in resting state cells is below detection level; upregulated only in activated astrocytes; colocalizes with GLAST
Serpin G1	plasma protease C1 inhibitor	interferes with C3 and CFB by physical binding influencing the alternate complement activation, increased in LPS activation
TIMP1	tissue inhibitor of metalloproteinase 1	forms complexes with MMPs irreversibly inactivating them; able to induce reactive astrogliosis; produced by astrocytes in response to pathophysiological threat
TN-C	tenascin	extracellular matrix protein; engaged in astroglia development and synaptic plasticity, marker of reactive astrocytes in stroke, neuronal injury or glial scar formation, induced by MCAO
TSP-1	thrombospondin-1	STAT-3-regulated factor; stimulates synaptogenesis, found upregulated after motor neuron injury along with GFAP
**Intracellular Proteins**		
ALDOC	fructose-bisphosphate aldolase C	glycolytic enzyme; upregulated after spinal or brain injuries
ARG1	arginase 1	hydrolase; enzyme converting L-arginine in urea cycle, expressed in microglia but also in some astrocytes
FABP7	fatty acid-binding protein	expressed by injured astrocytes and is specific to them
GBP2	guanylate-binding protein 2	transducer; reduces GTP by hydrolyzation, anti-viral properties
HSPB1/HSP27	heat shock factor-binding protein 1	chaperones; overexpressed in neurodegenerative diseases
HSPB5	alpha-B crystallin	chaperones; overexpressed in neurodegenerative diseases
iNOS	inducible NO synthetase	cytoplasmic enzyme; engaged in cytokine-induced NO production during inflammatory response, strongly upregulated not only in microglia but also in reactive astrocytes
MAO-B	monoamine oxidase B	increase is a marker in neurodegenerative conditions affecting neurons releasing monoamines
NDRG2	N-Myc downstream-regulated gene 2	developmental protein; tumor suppressor and cellular stress-related protein associated with cell proliferation and differentiation; exclusively expressed by astrocytes
NFAT	nuclear factor of activated T cells	transcription factor
NRF2	nuclear factor erythroid 2-related factor 2	transcription factor; regulates gene expression related to oxidative stress and redox reactions; expressed exclusively in astrocytes in course of Parkinson’s disease, but additionally in microglia and neurons in Alzheimer’s disease
PTGS2	prostaglandin G/H synthase 2	responsible for stimulation of prostaglandin production; expression increased in injury and inflammation-related reactive astrogliosis
S100β	protein S100-B	cytosolic Ca^++^-binding protein; labels cell bodies of small astrocytes with less extended branching; preferentially marks gray matter cells complementing GFAP staining; actively secreted from astrocytes during cellular stress along with downregulation of its expression intracellularly
SOX9	SRY-box transcription factor 9	almost exclusively expressed by astrocytes in adult human CNS, strong upregulation reported in rodent ALS model
SPHK1	sphingosine kinase-1	catalyzes phosphorylation of sphingosine activating NFκB signaling and IL-17 secretion
STAT3	signal transducer and activator of transcription 3	JAK-STAT pathway element; necessary for astroglial differentiation; overexpressed by astroglia in injury-induced inflammation, but expressed by other cells
